# How to encourage patients with implanted electronic cardiac devices to participate in remote monitoring and follow-up?

**DOI:** 10.1007/s12471-014-0596-y

**Published:** 2014-09-04

**Authors:** N.M. van Hemel

**Affiliations:** Department of Cardiology, University Medical Center Utrecht, Heidelberglaan 100, 3584 CX Utrecht, the Netherlands

Around 1904 remote monitoring of electrical cardiac activity was carried out for the first time by Willem Einthoven: he recorded the one-lead ECG of a patient admitted to the Leiden University Clinic in his Physiology Laboratory at a distance of 1.5 km from the hospital. Rightly he called this recording the tele-cardiogram (1). Hundred years later remote monitoring has expanded markedly by tremendous technical achievements, information technology, and the worldwide internet, components that underlie the chain between the cardiac implanted electronic device (CIED), the patient at home or outdoors, and the hospital staff responsible for technical controls. The first series of successive outcome studies indicates that remote monitoring and follow-up either at regular periods replacing the in-hospital technical controls (2), or 24-h monitoring to detect timely device failures is safe, highly reliable, prompts replies to failures, and reduces the incidence of inappropriate ICD shocks. Thus remote follow-up and monitoring can be classified as a valuable additional tool for patient care. Afterwards several randomised studies underscored this favourable outcome whereas moderate cost saving (3, 4) could be demonstrated. Consequently a consensus document for remote CIED monitoring was composed for the Dutch cardiology profession that can be applied in daily practice (5).

Despite the favourable messages of these papers, several obstacles block the employment of CIED remote monitoring in Western general practice. The overwhelming amount of data that can arrive at the desk of the cardiologist or allied professional 24 h a day/7 days a week (6) and wait for reply or action or not, together with the sometimes unknown potential clinical relevance of the automated generated plots and graphs are serious pitfalls. In addition, lack of justifiable and fair reimbursement, and of legal arrangements of various parties, and in particular the contested willingness of some CIED patients to participate in remote monitoring inhibit further employment of this remote care (7). However, several studies did show obvious patient satisfaction and acceptance of remote ICD follow-up (8), while younger patients and smaller clinics (7), and probably other physical and mental characteristics, are associated with noncompliance and preference for in-hospital checks.

In this issue Versteeg and co-workers present the rationale and design of the REMOTE-CIED study that attempts to establish the patient perspective on remote ICD monitoring which ‘could help to support patient-centred care in the future’ and to calculate cost effectiveness of this method (9). In this multicentre, prospective European study patients with a first ICD or CRT-ICD for primary or secondary prevention from fatal arrhythmias, and who have a left ventricular ejection fraction < 35 % and mild to moderate congestive heart failure, will be recruited (*n* = 900) and followed for > 2 years. To assess patient attitudes and perception of CIED remote monitoring, randomisation between in-hospital follow-up only or the combined patient remote follow-up and annual in-hospital control will be carried out. Several validated questionnaires have to be filled out at scheduled time intervals. Demographic, medical and CIED-related events and failures will be noted to determine factors associated with the perception of remote monitoring of the participants.

This study is specifically focused on patient feelings because according to the investigators: ‘previous trials have fallen short in their patient-centredness and have left an important question unanswered: namely what do patients think about remote monitoring?’ (9). This is indeed true because this subject was only studied in (small) outcome studies in selected patients (8) or the patient satisfaction with telemedicine was neither clearly defined nor assessed with validated methods (10). Because patient perception about remote CIED follow-up is without doubt strongly determined by human influences - of the cardiologist, allied professional or family-, the progress of the underlying heart disease, unsatisfying contribution of CRT to the failing cardiac muscle (in about 30 %), comorbidity and negative effects of ageing, inappropriate ICD treatment and so forth, the ultimate comparison of the results of both study arms can become a scientific puzzle and not a clear yes/no answer. This potential problem can be solved by a much longer follow-up of at least 5 years that better reflects real life. Furthermore medicalisation and wish-fulfilling medicine evoked by remote monitoring and follow-up are not addressed at all in this study. Attention to these potential side effects is justified because the Western health care system is under pressure. Third, because the study will be conducted in five European countries assessment of cost-effectiveness is complex and confusing due to large variations in cost calculations of all care components in the participating countries. This part of the objectives has already been sufficiently covered showing clear cost saving in most countries (3, 4).

Despite these obstacles, this study warrants implementation and participation of Dutch hospitals with a CIED license and experience because we eventually want to get validated insight into the characteristics of patients who will most profit or not from CIED remote monitoring. These data are strongly desired to also convince health insurance companies that remote ICD follow-up justifies reimbursement and promotes sustainable care with high and measurable quality. In the meantime we go forwards to encourage our CIED patients to profit from remote technical follow-up with the annual medical visit in stable patients while care of heart failure patients with CIED is routinely covered in the specific outpatient departments. In conclusion, we need more and detailed facts to convince ICD and CRT-ICD recipients that remote follow-up is worthwhile and that the patient is more than a computer file (7).
